# Crystal structure of (*E*)-1-[4-({4-[(4-meth­oxy­benzyl­idene)amino]­phen­yl}sulfan­yl)phen­yl]ethan-1-one

**DOI:** 10.1107/S205698901500033X

**Published:** 2015-01-14

**Authors:** Rabihe Hebbachi, Amel Djedouani, Soumia Kadri, Hénia Mousser, Abdelhamid Mousser

**Affiliations:** a Département de Chimie, Faculté des Sciences Exactes, Université de Constantine 1, Route de Ain El Bey, Constantine, Algeria; b Ecole Normale Supérieure de Constantine, Ville Universitaire Ali Mendjeli, Constantine, Algeria; c Laboratoire de Physicochimie Analytique et Cristallochimie de Matériaux Organométalliques et Biomoléculaires, Université de Constantine 1, Constantine, Algeria

**Keywords:** crystal structure, Schiff base, 4-amino-4-acetyl­diphenyl sulfide, C—H⋯π inter­actions

## Abstract

The title Schiff base compound, C_22_H_19_NO_2_S, crystallized with two independent mol­ecules (*A* and *B*) in the asymmetric unit. Both mol­ecules have an *E* conformation about the C=N bond. The two mol­ecules differ in the orientation of the aromatic rings with respect to each other. The outer 4-meth­oxy­benzene ring is inclined to the central benzene ring and the outer 4-acetyl­benzene ring by 1.80 (19) and 63.73 (19)°, respectively, in mol­ecule *A*, and by 6.72 (18) and 68.53 (19)°, respectively, in mol­ecule *B*. The two outer benzene rings are inclined to one another by 63.77 (18) and 63.19 (18)° in mol­ecules *A* and *B*, respectively. In the crystal, the individual mol­ecules stack in columns along [010], and are linked by a number of C—H⋯π inter­actions, forming slabs lying parallel to (001).

## Related literature   

For the synthesis and structures of Schiff bases, see, for example: Kahwa *et al.* (1986 ▸). For their use as protein and enzyme mimics, see: Santos *et al.* (2001 ▸). For their use as corrosion inhibitors, see: Ahamad *et al.* (2010 ▸); Negm *et al.* (2010 ▸). For their coordination properties, see: Özkar *et al.* (2004 ▸); Hebbachi & Benali-Cherif (2005 ▸). For complexation of Schiff bases with transition metals, see: Izatt *et al.* (1995 ▸); Kalcher *et al.* (1995 ▸). For the crystal structure of a very similar Schiff base compound derived from 4-amino-4-acetyl­diphenyl sulfide, see: Hebbachi *et al.* (2013 ▸).
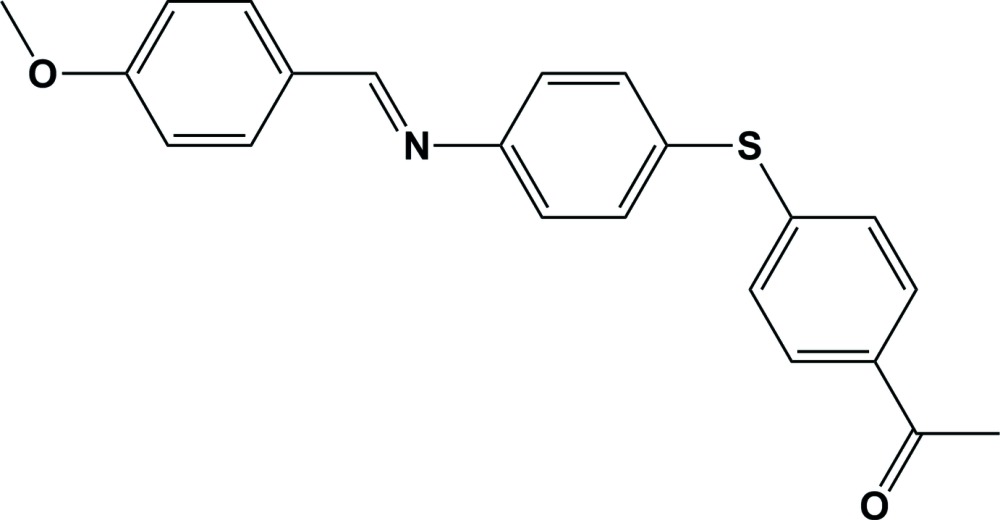


## Experimental   

### Crystal data  

C_22_H_19_NO_2_S
*M* *_r_* = 361.44
Triclinic, 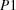

*a* = 5.7708 (2) Å
*b* = 8.0867 (3) Å
*c* = 19.6929 (8) Å
α = 81.844 (2)°
β = 86.664 (3)°
γ = 85.662 (3)°
*V* = 906.05 (6) Å^3^
*Z* = 2
Mo *K*α radiation
μ = 0.20 mm^−1^
*T* = 293 K
0.1 × 0.1 × 0.1 mm

### Data collection  

Bruker SMART 1K CCD area-detector diffractometer
19586 measured reflections
6013 independent reflections
4850 reflections with *I* > 2σ(*I*)
*R*_int_ = 0.032

### Refinement  

*R*[*F* ^2^ > 2σ(*F* ^2^)] = 0.039
*wR*(*F* ^2^) = 0.076
*S* = 1.03
6013 reflections
473 parameters
3 restraints
H-atom parameters constrained
Δρ_max_ = 0.14 e Å^−3^
Δρ_min_ = −0.19 e Å^−3^
Absolute structure: Flack *x* determined using 1952 quotients [(*I* ^+^)−(*I* ^−^)]/[(*I* ^+^)+(*I* ^−^)] (Parsons *et al.*, 2013 ▸)
Absolute structure parameter: 0.06 (3)

### 

Data collection: *SMART* (Bruker, 2006 ▸); cell refinement: *SAINT* (Bruker, 2006 ▸); data reduction: *SAINT*; program(s) used to solve structure: *SHELXS97* (Sheldrick, 2008 ▸); program(s) used to refine structure: *SHELXL2014* (Sheldrick, 2015 ▸); molecular graphics: *ORTEP-3 for Windows* (Farrugia, 2012 ▸) and *Mercury* (Macrae *et al.*, 2008 ▸); software used to prepare material for publication: *WinGX* (Farrugia, 2012 ▸) and *SHELXL2014*.

## Supplementary Material

Crystal structure: contains datablock(s) I, Global. DOI: 10.1107/S205698901500033X/su5056sup1.cif

Structure factors: contains datablock(s) I. DOI: 10.1107/S205698901500033X/su5056Isup2.hkl

Supporting information file. DOI: 10.1107/S205698901500033X/su5056Isup3.cml

**Fig. 1.**
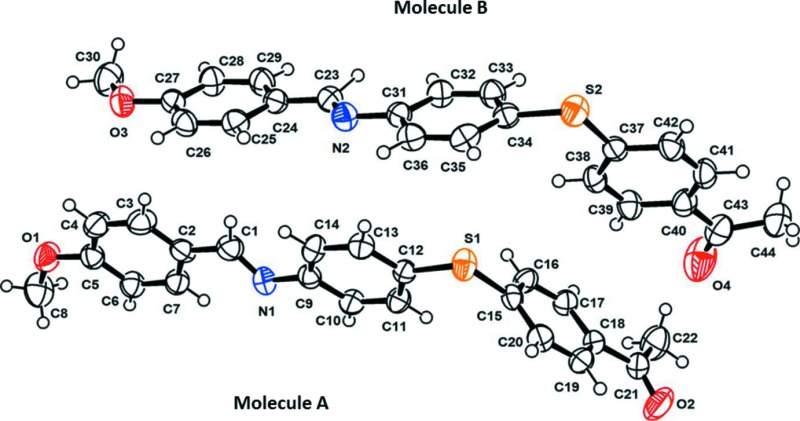
The molecular structure of the two independent molecules (A and B) of the title compound, with atom labelling. Displacement ellipsoids are drawn at the 50% probability level.

**Fig. 2.**
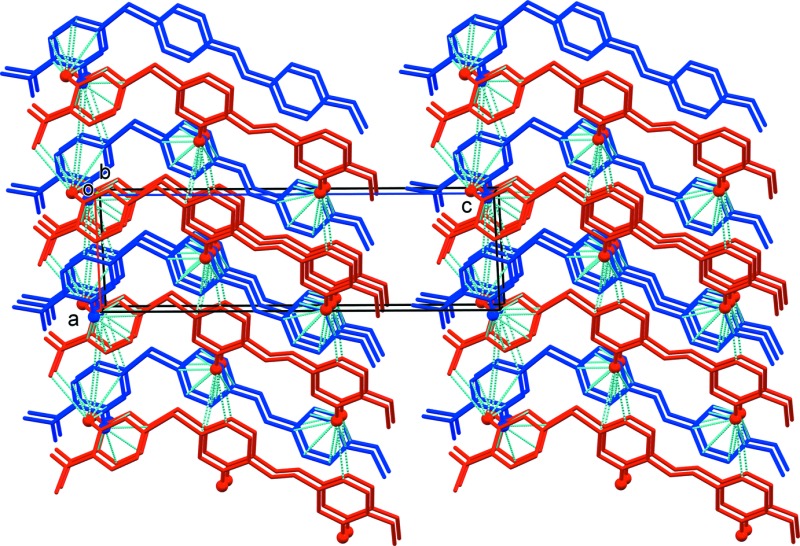
A view along the b axis of the crystal packing of the title compound. C-H···π interactions are shown as dashed lines (see Table 1 for details; molecule A is red; molecule B is blue; H atoms not involved in these interactions have been omitted for clarity).

. DOI: 10.1107/S205698901500033X/su5056fig1.tif
The mol­ecular structure of the two independent mol­ecules (A and B) of the title compound, with atom labelling. Displacement ellipsoids are drawn at the 50% probability level.

b . DOI: 10.1107/S205698901500033X/su5056fig2.tif
A view along the *b* axis of the crystal packing of the title compound. C-H⋯π inter­actions are shown as dashed lines (see Table 1 for details; mol­ecule A is red; mol­ecule B is blue; H atoms not involved in these inter­actions have been omitted for clarity).

CCDC reference: 1042562

Additional supporting information:  crystallographic information; 3D view; checkCIF report

## Figures and Tables

**Table 1:** Hydrogen-bond geometry (, ) *Cg*1, *Cg*2, *Cg*3 and *Cg*6 are the centroids of the C2C7, C9C14, C15C20 and C37C42 rings, respectively.

*D*H*A*	*D*H	H*A*	*D* *A*	*D*H*A*
C17H17*Cg*6^i^	0.93	3.00	3.734(4)	137
C26H26*Cg*1	0.93	2.96	3.763(4)	146
C32H32*Cg*2	0.93	2.98	3.706(4)	136
C41H41*Cg*3^ii^	0.93	2.99	3.670(4)	131

Symmetry codes: (i) 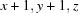; (ii) 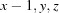.

## supplementary crystallographic information

### S1. Comment

The synthesis and structures of Schiff bases have attracted much attention in biology and chemistry (Kahwa *et al.*, 1986). One of the aims of investigating their structural chemistry is to develop protein and enzyme mimics (Santos *et al.*, 2001). Structural information is useful in investigating the coordination properties of Schiff bases functioning as ligands (Özkar *et al.*, 2004; Hebbachi & Benali-Cherif, 2005). They have a great capacity for complexation of transition metals (Izatt *et al.*, 1995; Kalcher *et al.*, 1995). They are also used as corrosion inhibitors (Ahamad *et al.*, 2010; Negm *et al.*, 2010). There are only a few reported crystal structures of Schiff bases derived from 4-amino-4-acetyldiphenyl sulfide (Hebbachi *et al.*, 2013). As a part of our ongoing research, we have synthesized the title compound and report herein on its crystal structure.

The title compound, Fig. 1, crystallized with two independent molecules (A and B) in the asymmetric unit. Both molecules have an E conformation about the C═N bond, with torsion angles C2—C1═N1—C9 and C24—C23═ N2—C31 being -179.9 (3) and 177.2 (3), respectively.

The two molecules differ in the orientation of the aromatic rings with respect to one another. The outer 4-methoxybenzene ring is inclined to the central benzene ring and the outer 3-acetylbenzene ring by 1.80 (19) and 63.73 (19) °, respectively, in molecule A, and by 6.72 (18) and 68.53 (19) °, respectively in molecule B. The two outer benzene rings are inclined to one another by 63.77 (18) and 63.19 (18) ° in molecules A and B, respectively.

The bond lengths and angles are close to those observed for a very similar structure, viz. (*E*)-1-(4-((4-(((4-hydroxynaphthalen-1-yl)methylene)amino)phenyl)thio) phenyl)ethan-1-one (Hebbachi *et al.*, 2013). For example, the sulfur atom has *sp*^3^ hybridization as indicated by the C—S—C angle of 106.01 (15) and 105.99 (15) ° in molecules A and B, respectively, compared to 104.88 (15) ° observed in the above mentioned compound.

In the crystal, molecules stack along [010] in columns composed of either A or B molecules, and are linked by a number of C-H···π interactions (Table 1 and Fig. 2) forming slabs lying parallel to (001).

### S2. Experimental

The title Schiff base was prepared by condensation of 4-amino-4-acetyl diphenylsulfure and anisaldehyde in a 1:1 molar ratio, in an ethanol solution containing a few drops of dry piperidine. The mixture was stirred under reflux for 3 h. The mixture was then concentrated and cooled. Colourless prismatic crystals of title compound were obtained by recrystallization from a mixture of chloroform/hexane (1/1). They were collected by filtration and dried in air (yield: 64%; m.p.: 421 K).

### S3. Refinement

H atoms were positioned geometrically and refined using a riding model: C—H = 0.93 - 0.98 Å with *U*_iso_(H) = 1.5U_eq_(C) for methyl H atoms and = 1.2U_eq_(C) for other H atoms.

### Crystal data

**Table:**

C_22_H_19_NO_2_S	*Z* = 2
*M**_r_* = 361.44	*F*(000) = 380
Triclinic, *P*1	*D*_x_ = 1.325 Mg m^−^^3^
*a* = 5.7708 (2) Å	Mo *K*α radiation, λ = 0.71073 Å
*b* = 8.0867 (3) Å	θ = 1.0–27.1°
*c* = 19.6929 (8) Å	µ = 0.20 mm^−^^1^
α = 81.844 (2)°	*T* = 293 K
β = 86.664 (3)°	Prism, colourless
γ = 85.662 (3)°	0.1 × 0.1 × 0.1 mm
*V* = 906.05 (6) Å^3^	

### Data collection

**Table:**

Bruker SMART 1K CCD area-detector diffractometer	4850 reflections with *I* > 2σ(*I*)
Radiation source: fine-focus sealed tube	*R*_int_ = 0.032
Graphite monochromator	θ_max_ = 25.0°, θ_min_ = 2.6°
ω scan	*h* = −6→6
19586 measured reflections	*k* = −9→9
6013 independent reflections	*l* = −22→23

### Refinement

**Table:**

Refinement on *F*^2^	Hydrogen site location: inferred from neighbouring sites
Least-squares matrix: full	H-atom parameters constrained
*R*[*F*^2^ > 2σ(*F*^2^)] = 0.039	*w* = 1/[σ^2^(*F*_o_^2^) + (0.0319*P*)^2^ + 0.0416*P*] where *P* = (*F*_o_^2^ + 2*F*_c_^2^)/3
*wR*(*F*^2^) = 0.076	(Δ/σ)_max_ < 0.001
*S* = 1.03	Δρ_max_ = 0.14 e Å^−^^3^
6013 reflections	Δρ_min_ = −0.19 e Å^−^^3^
473 parameters	Absolute structure: Flack *x* determined using 1952 quotients [(*I*^+^)-(*I*^-^)]/[(*I*^+^)+(*I*^-^)] (Parsons *et al.*, 2013)
3 restraints	Absolute structure parameter: 0.06 (3)

### Special details

**Table:**

Geometry. All esds (except the esd in the dihedral angle between two l.s. planes) are estimated using the full covariance matrix. The cell esds are taken into account individually in the estimation of esds in distances, angles and torsion angles; correlations between esds in cell parameters are only used when they are defined by crystal symmetry. An approximate (isotropic) treatment of cell esds is used for estimating esds involving l.s. planes.

### Fractional atomic coordinates and isotropic or equivalent isotropic displacement parameters (Å^2^)

**Table:**

	*x*	*y*	*z*	*U*_iso_*/*U*_eq_	
S1	0.40421 (16)	0.84717 (13)	0.09943 (5)	0.0521 (3)	
O1	1.2589 (5)	0.9295 (3)	0.65028 (13)	0.0582 (8)	
O2	1.1549 (6)	1.1965 (5)	−0.14550 (16)	0.0981 (13)	
N1	0.7980 (6)	0.9227 (4)	0.36608 (16)	0.0474 (8)	
C1	0.9871 (7)	0.8570 (5)	0.38833 (19)	0.0467 (10)	
H1	1.0810	0.7913	0.3612	0.056*	
C2	1.0659 (6)	0.8797 (5)	0.45527 (18)	0.0410 (9)	
C3	0.9313 (7)	0.9756 (5)	0.49822 (19)	0.0473 (10)	
H3	0.7912	1.0293	0.4835	0.057*	
C4	1.0031 (7)	0.9914 (5)	0.5619 (2)	0.0474 (10)	
H4	0.9124	1.0569	0.5898	0.057*	
C5	1.2101 (7)	0.9106 (5)	0.58501 (18)	0.0418 (10)	
C6	1.3488 (7)	0.8174 (5)	0.54288 (19)	0.0513 (11)	
H6	1.4893	0.7645	0.5577	0.062*	
C7	1.2765 (7)	0.8037 (5)	0.4784 (2)	0.0519 (10)	
H7	1.3709	0.7422	0.4498	0.062*	
C8	1.4655 (8)	0.8448 (6)	0.6785 (2)	0.0705 (13)	
H8A	1.4721	0.8630	0.7255	0.106*	
H8B	1.5993	0.8877	0.6526	0.106*	
H8C	1.4638	0.7271	0.6764	0.106*	
C9	0.7237 (6)	0.9003 (4)	0.30085 (18)	0.0395 (9)	
C10	0.5119 (6)	0.9821 (5)	0.28283 (19)	0.0454 (10)	
H10	0.4293	1.0456	0.3132	0.054*	
C11	0.4219 (6)	0.9703 (5)	0.22036 (19)	0.0446 (10)	
H11	0.2796	1.0260	0.2089	0.054*	
C12	0.5420 (6)	0.8762 (5)	0.17482 (18)	0.0404 (9)	
C13	0.7546 (6)	0.7946 (5)	0.19237 (19)	0.0462 (10)	
H13	0.8370	0.7312	0.1619	0.055*	
C14	0.8444 (6)	0.8068 (5)	0.25442 (19)	0.0446 (10)	
H14	0.9876	0.7520	0.2655	0.054*	
C15	0.5884 (6)	0.9284 (4)	0.02990 (18)	0.0392 (9)	
C16	0.7859 (6)	1.0131 (4)	0.03509 (18)	0.0417 (9)	
H16	0.8307	1.0313	0.0779	0.050*	
C17	0.9151 (6)	1.0700 (5)	−0.02354 (19)	0.0474 (10)	
H17	1.0464	1.1273	−0.0196	0.057*	
C18	0.8547 (6)	1.0441 (5)	−0.08816 (19)	0.0456 (10)	
C19	0.6550 (7)	0.9614 (5)	−0.09213 (19)	0.0489 (10)	
H19	0.6093	0.9443	−0.1350	0.059*	
C20	0.5227 (7)	0.9041 (5)	−0.03450 (19)	0.0475 (10)	
H20	0.3894	0.8491	−0.0386	0.057*	
C21	1.0007 (8)	1.1056 (6)	−0.1499 (2)	0.0569 (11)	
C22	0.9573 (9)	1.0531 (6)	−0.2177 (2)	0.0800 (15)	
H22A	1.0659	1.1021	−0.2522	0.120*	
H22B	0.8016	1.0901	−0.2302	0.120*	
H22C	0.9767	0.9333	−0.2144	0.120*	
S2	−0.10116 (16)	0.32763 (13)	0.16371 (5)	0.0510 (3)	
O3	0.8434 (5)	0.4410 (4)	0.69853 (14)	0.0654 (8)	
O4	0.2586 (6)	0.6780 (4)	−0.14986 (14)	0.0845 (10)	
N2	0.4418 (5)	0.4400 (4)	0.40143 (16)	0.0475 (8)	
C23	0.4230 (7)	0.3483 (5)	0.4586 (2)	0.0528 (11)	
H23	0.3251	0.2609	0.4627	0.063*	
C24	0.5470 (7)	0.3721 (5)	0.51887 (19)	0.0446 (10)	
C25	0.7361 (7)	0.4704 (5)	0.5146 (2)	0.0490 (10)	
H25	0.7923	0.5194	0.4718	0.059*	
C26	0.8425 (7)	0.4964 (5)	0.57344 (19)	0.0487 (10)	
H26	0.9686	0.5627	0.5701	0.058*	
C27	0.7591 (7)	0.4226 (5)	0.6370 (2)	0.0466 (10)	
C28	0.5749 (7)	0.3232 (5)	0.6414 (2)	0.0539 (11)	
H28	0.5203	0.2723	0.6840	0.065*	
C29	0.4705 (7)	0.2986 (5)	0.5829 (2)	0.0557 (11)	
H29	0.3458	0.2310	0.5866	0.067*	
C30	1.0244 (8)	0.5508 (6)	0.6994 (2)	0.0753 (14)	
H30A	1.0722	0.5461	0.7456	0.113*	
H30B	0.9687	0.6632	0.6826	0.113*	
H30C	1.1544	0.5171	0.6706	0.113*	
C31	0.3062 (6)	0.4115 (4)	0.34626 (18)	0.0399 (9)	
C32	0.3869 (6)	0.4741 (4)	0.28040 (18)	0.0429 (9)	
H32	0.5214	0.5320	0.2746	0.052*	
C33	0.2704 (6)	0.4515 (5)	0.22338 (19)	0.0422 (9)	
H33	0.3268	0.4939	0.1797	0.051*	
C34	0.0701 (6)	0.3659 (4)	0.23136 (17)	0.0362 (9)	
C35	−0.0163 (6)	0.3070 (5)	0.29683 (18)	0.0428 (10)	
H35	−0.1530	0.2516	0.3025	0.051*	
C36	0.1005 (6)	0.3307 (5)	0.35358 (18)	0.0455 (10)	
H36	0.0405	0.2920	0.3973	0.055*	
C37	0.0526 (6)	0.4036 (4)	0.08628 (17)	0.0385 (9)	
C38	0.2689 (6)	0.3346 (5)	0.06659 (18)	0.0438 (9)	
H38	0.3481	0.2541	0.0969	0.053*	
C39	0.3663 (7)	0.3849 (5)	0.00259 (19)	0.0434 (10)	
H39	0.5111	0.3369	−0.0101	0.052*	
C40	0.2549 (6)	0.5052 (5)	−0.04367 (17)	0.0396 (9)	
C41	0.0402 (7)	0.5757 (5)	−0.02320 (19)	0.0476 (10)	
H41	−0.0369	0.6583	−0.0531	0.057*	
C42	−0.0611 (7)	0.5250 (5)	0.04106 (19)	0.0465 (10)	
H42	−0.2059	0.5726	0.0539	0.056*	
C43	0.3520 (7)	0.5607 (5)	−0.1144 (2)	0.0515 (10)	
C44	0.5658 (8)	0.4716 (6)	−0.1407 (2)	0.0737 (14)	
H44A	0.5995	0.5168	−0.1877	0.111*	
H44B	0.5421	0.3545	−0.1378	0.111*	
H44C	0.6939	0.4859	−0.1136	0.111*	

### Atomic displacement parameters (Å^2^)

**Table:**

	*U*^11^	*U*^22^	*U*^33^	*U*^12^	*U*^13^	*U*^23^
S1	0.0437 (6)	0.0641 (8)	0.0497 (6)	−0.0147 (5)	−0.0073 (5)	−0.0046 (5)
O1	0.064 (2)	0.069 (2)	0.0441 (17)	−0.0068 (16)	−0.0058 (15)	−0.0138 (14)
O2	0.085 (3)	0.149 (4)	0.062 (2)	−0.049 (3)	0.0066 (19)	−0.001 (2)
N1	0.046 (2)	0.052 (2)	0.0449 (19)	−0.0003 (17)	−0.0026 (17)	−0.0098 (16)
C1	0.053 (3)	0.044 (3)	0.043 (2)	0.002 (2)	0.002 (2)	−0.0083 (18)
C2	0.042 (2)	0.040 (2)	0.040 (2)	−0.0033 (18)	0.0031 (19)	−0.0058 (18)
C3	0.044 (2)	0.046 (3)	0.051 (3)	0.0025 (19)	0.001 (2)	−0.007 (2)
C4	0.048 (3)	0.047 (3)	0.048 (2)	−0.002 (2)	0.008 (2)	−0.0152 (19)
C5	0.047 (3)	0.041 (2)	0.039 (2)	−0.011 (2)	−0.002 (2)	−0.0073 (18)
C6	0.046 (2)	0.060 (3)	0.047 (2)	0.005 (2)	−0.007 (2)	−0.011 (2)
C7	0.053 (3)	0.053 (3)	0.050 (2)	0.006 (2)	0.000 (2)	−0.014 (2)
C8	0.073 (3)	0.085 (4)	0.056 (3)	−0.014 (3)	−0.017 (3)	−0.006 (2)
C9	0.039 (2)	0.039 (2)	0.040 (2)	−0.0044 (18)	0.0025 (19)	−0.0066 (17)
C10	0.041 (2)	0.044 (3)	0.050 (2)	0.0023 (19)	0.003 (2)	−0.0083 (19)
C11	0.033 (2)	0.047 (3)	0.050 (3)	0.0010 (18)	−0.001 (2)	0.0019 (19)
C12	0.040 (2)	0.040 (2)	0.040 (2)	−0.0035 (19)	−0.0002 (19)	−0.0033 (18)
C13	0.045 (3)	0.049 (3)	0.046 (2)	0.002 (2)	−0.001 (2)	−0.0119 (19)
C14	0.039 (2)	0.045 (3)	0.048 (2)	0.0059 (19)	−0.006 (2)	−0.006 (2)
C15	0.041 (2)	0.037 (2)	0.041 (2)	0.0025 (18)	−0.0080 (18)	−0.0080 (17)
C16	0.041 (2)	0.047 (2)	0.040 (2)	−0.0034 (19)	−0.0093 (19)	−0.0110 (18)
C17	0.040 (2)	0.059 (3)	0.046 (2)	−0.009 (2)	−0.006 (2)	−0.011 (2)
C18	0.045 (2)	0.049 (3)	0.042 (2)	0.006 (2)	−0.0108 (19)	−0.0064 (19)
C19	0.054 (3)	0.055 (3)	0.039 (2)	−0.001 (2)	−0.015 (2)	−0.0109 (19)
C20	0.047 (3)	0.050 (3)	0.048 (3)	−0.004 (2)	−0.015 (2)	−0.012 (2)
C21	0.054 (3)	0.065 (3)	0.050 (3)	0.005 (2)	−0.005 (2)	−0.003 (2)
C22	0.114 (4)	0.078 (4)	0.047 (3)	0.001 (3)	−0.002 (3)	−0.010 (2)
S2	0.0423 (6)	0.0712 (8)	0.0421 (6)	−0.0166 (5)	−0.0022 (5)	−0.0097 (5)
O3	0.078 (2)	0.073 (2)	0.0477 (18)	−0.0194 (17)	−0.0165 (16)	−0.0049 (15)
O4	0.118 (3)	0.082 (2)	0.0437 (18)	0.014 (2)	−0.0032 (18)	0.0140 (17)
N2	0.051 (2)	0.053 (2)	0.041 (2)	−0.0030 (16)	−0.0040 (17)	−0.0137 (17)
C23	0.059 (3)	0.045 (3)	0.056 (3)	−0.007 (2)	−0.011 (2)	−0.008 (2)
C24	0.051 (3)	0.037 (2)	0.049 (2)	−0.001 (2)	−0.011 (2)	−0.0106 (19)
C25	0.052 (3)	0.051 (3)	0.043 (2)	−0.001 (2)	0.002 (2)	−0.0062 (19)
C26	0.047 (2)	0.053 (3)	0.047 (3)	−0.009 (2)	−0.004 (2)	−0.008 (2)
C27	0.050 (3)	0.048 (3)	0.044 (2)	−0.001 (2)	−0.008 (2)	−0.0105 (19)
C28	0.061 (3)	0.054 (3)	0.046 (2)	−0.008 (2)	−0.009 (2)	0.000 (2)
C29	0.058 (3)	0.050 (3)	0.059 (3)	−0.013 (2)	−0.012 (2)	0.001 (2)
C30	0.066 (3)	0.097 (4)	0.070 (3)	−0.024 (3)	−0.018 (3)	−0.021 (3)
C31	0.041 (2)	0.039 (2)	0.040 (2)	0.0026 (18)	−0.0019 (18)	−0.0089 (18)
C32	0.038 (2)	0.046 (3)	0.045 (2)	−0.0111 (18)	−0.0022 (19)	−0.0047 (18)
C33	0.040 (2)	0.048 (2)	0.037 (2)	−0.0061 (18)	0.0042 (18)	−0.0023 (17)
C34	0.034 (2)	0.038 (2)	0.036 (2)	0.0002 (18)	−0.0014 (17)	−0.0065 (17)
C35	0.036 (2)	0.048 (3)	0.045 (2)	−0.0100 (18)	0.0006 (19)	−0.0041 (19)
C36	0.044 (2)	0.057 (3)	0.035 (2)	−0.003 (2)	0.0018 (19)	−0.0033 (19)
C37	0.040 (2)	0.042 (2)	0.036 (2)	−0.0046 (18)	−0.0063 (18)	−0.0107 (18)
C38	0.043 (2)	0.045 (2)	0.040 (2)	0.0057 (19)	−0.0080 (19)	0.0012 (17)
C39	0.041 (2)	0.047 (3)	0.042 (2)	0.0021 (19)	−0.0037 (19)	−0.0063 (19)
C40	0.047 (2)	0.039 (2)	0.035 (2)	−0.0056 (18)	−0.0084 (18)	−0.0067 (17)
C41	0.054 (3)	0.045 (3)	0.044 (2)	0.005 (2)	−0.016 (2)	−0.0025 (18)
C42	0.041 (2)	0.049 (3)	0.049 (2)	0.0074 (19)	−0.007 (2)	−0.011 (2)
C43	0.069 (3)	0.050 (3)	0.037 (2)	−0.009 (2)	−0.010 (2)	−0.006 (2)
C44	0.073 (3)	0.092 (4)	0.053 (3)	−0.004 (3)	0.013 (3)	−0.005 (3)

### Geometric parameters (Å, º)

**Table:**

S1—C15	1.765 (4)	S2—C37	1.775 (3)
S1—C12	1.776 (4)	S2—C34	1.779 (3)
O1—C5	1.363 (4)	O3—C27	1.362 (4)
O1—C8	1.430 (5)	O3—C30	1.424 (5)
O2—C21	1.210 (5)	O4—C43	1.207 (5)
N1—C1	1.252 (4)	N2—C23	1.260 (4)
N1—C9	1.417 (4)	N2—C31	1.429 (4)
C1—C2	1.459 (5)	C23—C24	1.464 (5)
C1—H1	0.9300	C23—H23	0.9300
C2—C7	1.391 (5)	C24—C29	1.378 (5)
C2—C3	1.392 (5)	C24—C25	1.389 (5)
C3—C4	1.369 (5)	C25—C26	1.391 (5)
C3—H3	0.9300	C25—H25	0.9300
C4—C5	1.386 (5)	C26—C27	1.385 (5)
C4—H4	0.9300	C26—H26	0.9300
C5—C6	1.381 (5)	C27—C28	1.372 (5)
C6—C7	1.380 (5)	C28—C29	1.376 (5)
C6—H6	0.9300	C28—H28	0.9300
C7—H7	0.9300	C29—H29	0.9300
C8—H8A	0.9600	C30—H30A	0.9600
C8—H8B	0.9600	C30—H30B	0.9600
C8—H8C	0.9600	C30—H30C	0.9600
C9—C10	1.385 (5)	C31—C36	1.388 (5)
C9—C14	1.391 (5)	C31—C32	1.391 (5)
C10—C11	1.381 (5)	C32—C33	1.382 (5)
C10—H10	0.9300	C32—H32	0.9300
C11—C12	1.381 (5)	C33—C34	1.382 (5)
C11—H11	0.9300	C33—H33	0.9300
C12—C13	1.386 (5)	C34—C35	1.387 (5)
C13—C14	1.374 (5)	C35—C36	1.382 (5)
C13—H13	0.9300	C35—H35	0.9300
C14—H14	0.9300	C36—H36	0.9300
C15—C20	1.389 (5)	C37—C42	1.382 (5)
C15—C16	1.389 (5)	C37—C38	1.385 (5)
C16—C17	1.379 (5)	C38—C39	1.370 (5)
C16—H16	0.9300	C38—H38	0.9300
C17—C18	1.387 (5)	C39—C40	1.383 (5)
C17—H17	0.9300	C39—H39	0.9300
C18—C19	1.387 (5)	C40—C41	1.386 (5)
C18—C21	1.486 (5)	C40—C43	1.491 (5)
C19—C20	1.374 (5)	C41—C42	1.384 (5)
C19—H19	0.9300	C41—H41	0.9300
C20—H20	0.9300	C42—H42	0.9300
C21—C22	1.498 (6)	C43—C44	1.485 (6)
C22—H22A	0.9600	C44—H44A	0.9600
C22—H22B	0.9600	C44—H44B	0.9600
C22—H22C	0.9600	C44—H44C	0.9600
			
C15—S1—C12	105.80 (17)	C37—S2—C34	105.96 (16)
C5—O1—C8	118.5 (3)	C27—O3—C30	118.7 (3)
C1—N1—C9	121.9 (3)	C23—N2—C31	119.6 (3)
N1—C1—C2	122.5 (3)	N2—C23—C24	123.6 (4)
N1—C1—H1	118.8	N2—C23—H23	118.2
C2—C1—H1	118.8	C24—C23—H23	118.2
C7—C2—C3	118.1 (4)	C29—C24—C25	118.2 (3)
C7—C2—C1	120.8 (3)	C29—C24—C23	119.2 (4)
C3—C2—C1	121.1 (3)	C25—C24—C23	122.6 (4)
C4—C3—C2	120.6 (4)	C24—C25—C26	120.9 (4)
C4—C3—H3	119.7	C24—C25—H25	119.5
C2—C3—H3	119.7	C26—C25—H25	119.5
C3—C4—C5	120.5 (4)	C27—C26—C25	119.4 (4)
C3—C4—H4	119.7	C27—C26—H26	120.3
C5—C4—H4	119.7	C25—C26—H26	120.3
O1—C5—C6	124.7 (4)	O3—C27—C28	114.8 (4)
O1—C5—C4	115.4 (3)	O3—C27—C26	125.4 (4)
C6—C5—C4	119.9 (3)	C28—C27—C26	119.8 (4)
C7—C6—C5	119.2 (4)	C27—C28—C29	120.3 (4)
C7—C6—H6	120.4	C27—C28—H28	119.9
C5—C6—H6	120.4	C29—C28—H28	119.9
C6—C7—C2	121.6 (4)	C28—C29—C24	121.4 (4)
C6—C7—H7	119.2	C28—C29—H29	119.3
C2—C7—H7	119.2	C24—C29—H29	119.3
O1—C8—H8A	109.5	O3—C30—H30A	109.5
O1—C8—H8B	109.5	O3—C30—H30B	109.5
H8A—C8—H8B	109.5	H30A—C30—H30B	109.5
O1—C8—H8C	109.5	O3—C30—H30C	109.5
H8A—C8—H8C	109.5	H30A—C30—H30C	109.5
H8B—C8—H8C	109.5	H30B—C30—H30C	109.5
C10—C9—C14	118.5 (3)	C36—C31—C32	118.3 (3)
C10—C9—N1	115.5 (3)	C36—C31—N2	125.3 (3)
C14—C9—N1	126.0 (3)	C32—C31—N2	116.4 (3)
C11—C10—C9	120.8 (3)	C33—C32—C31	121.1 (3)
C11—C10—H10	119.6	C33—C32—H32	119.5
C9—C10—H10	119.6	C31—C32—H32	119.5
C12—C11—C10	120.3 (3)	C34—C33—C32	120.0 (3)
C12—C11—H11	119.8	C34—C33—H33	120.0
C10—C11—H11	119.8	C32—C33—H33	120.0
C11—C12—C13	119.2 (3)	C33—C34—C35	119.6 (3)
C11—C12—S1	118.3 (3)	C33—C34—S2	125.6 (3)
C13—C12—S1	122.2 (3)	C35—C34—S2	114.7 (3)
C14—C13—C12	120.4 (3)	C36—C35—C34	120.0 (4)
C14—C13—H13	119.8	C36—C35—H35	120.0
C12—C13—H13	119.8	C34—C35—H35	120.0
C13—C14—C9	120.7 (3)	C35—C36—C31	120.9 (3)
C13—C14—H14	119.6	C35—C36—H36	119.5
C9—C14—H14	119.6	C31—C36—H36	119.5
C20—C15—C16	119.3 (3)	C42—C37—C38	119.2 (3)
C20—C15—S1	115.1 (3)	C42—C37—S2	117.6 (3)
C16—C15—S1	125.5 (3)	C38—C37—S2	122.8 (3)
C17—C16—C15	119.7 (3)	C39—C38—C37	120.1 (3)
C17—C16—H16	120.2	C39—C38—H38	120.0
C15—C16—H16	120.2	C37—C38—H38	120.0
C16—C17—C18	121.8 (3)	C38—C39—C40	121.7 (3)
C16—C17—H17	119.1	C38—C39—H39	119.2
C18—C17—H17	119.1	C40—C39—H39	119.2
C17—C18—C19	117.5 (3)	C39—C40—C41	117.9 (3)
C17—C18—C21	120.0 (4)	C39—C40—C43	123.4 (3)
C19—C18—C21	122.5 (3)	C41—C40—C43	118.7 (3)
C20—C19—C18	121.8 (3)	C42—C41—C40	121.0 (3)
C20—C19—H19	119.1	C42—C41—H41	119.5
C18—C19—H19	119.1	C40—C41—H41	119.5
C19—C20—C15	119.9 (4)	C37—C42—C41	120.1 (3)
C19—C20—H20	120.1	C37—C42—H42	119.9
C15—C20—H20	120.1	C41—C42—H42	119.9
O2—C21—C18	120.4 (4)	O4—C43—C44	120.1 (4)
O2—C21—C22	120.1 (4)	O4—C43—C40	120.3 (4)
C18—C21—C22	119.5 (4)	C44—C43—C40	119.6 (4)
C21—C22—H22A	109.5	C43—C44—H44A	109.5
C21—C22—H22B	109.5	C43—C44—H44B	109.5
H22A—C22—H22B	109.5	H44A—C44—H44B	109.5
C21—C22—H22C	109.5	C43—C44—H44C	109.5
H22A—C22—H22C	109.5	H44A—C44—H44C	109.5
H22B—C22—H22C	109.5	H44B—C44—H44C	109.5
			
C9—N1—C1—C2	179.7 (3)	C31—N2—C23—C24	−177.4 (3)
N1—C1—C2—C7	179.5 (4)	N2—C23—C24—C29	162.3 (4)
N1—C1—C2—C3	0.6 (6)	N2—C23—C24—C25	−15.8 (6)
C7—C2—C3—C4	−1.2 (6)	C29—C24—C25—C26	−1.2 (6)
C1—C2—C3—C4	177.8 (3)	C23—C24—C25—C26	176.9 (4)
C2—C3—C4—C5	−0.8 (5)	C24—C25—C26—C27	0.3 (6)
C8—O1—C5—C6	−1.3 (5)	C30—O3—C27—C28	−175.9 (4)
C8—O1—C5—C4	177.8 (4)	C30—O3—C27—C26	3.3 (6)
C3—C4—C5—O1	−177.2 (3)	C25—C26—C27—O3	−178.3 (3)
C3—C4—C5—C6	2.0 (6)	C25—C26—C27—C28	0.8 (6)
O1—C5—C6—C7	178.0 (4)	O3—C27—C28—C29	178.3 (4)
C4—C5—C6—C7	−1.2 (6)	C26—C27—C28—C29	−0.9 (6)
C5—C6—C7—C2	−0.9 (6)	C27—C28—C29—C24	0.0 (6)
C3—C2—C7—C6	2.0 (6)	C25—C24—C29—C28	1.1 (6)
C1—C2—C7—C6	−177.0 (4)	C23—C24—C29—C28	−177.1 (4)
C1—N1—C9—C10	−179.3 (4)	C23—N2—C31—C36	22.6 (5)
C1—N1—C9—C14	0.0 (5)	C23—N2—C31—C32	−159.0 (4)
C14—C9—C10—C11	0.4 (5)	C36—C31—C32—C33	−2.3 (5)
N1—C9—C10—C11	179.7 (3)	N2—C31—C32—C33	179.2 (3)
C9—C10—C11—C12	0.1 (5)	C31—C32—C33—C34	0.1 (5)
C10—C11—C12—C13	−0.4 (5)	C32—C33—C34—C35	1.8 (5)
C10—C11—C12—S1	173.4 (3)	C32—C33—C34—S2	179.4 (3)
C15—S1—C12—C11	121.4 (3)	C37—S2—C34—C33	5.3 (4)
C15—S1—C12—C13	−65.0 (3)	C37—S2—C34—C35	−176.9 (3)
C11—C12—C13—C14	0.2 (5)	C33—C34—C35—C36	−1.5 (5)
S1—C12—C13—C14	−173.3 (3)	S2—C34—C35—C36	−179.4 (3)
C12—C13—C14—C9	0.3 (6)	C34—C35—C36—C31	−0.7 (5)
C10—C9—C14—C13	−0.6 (5)	C32—C31—C36—C35	2.6 (5)
N1—C9—C14—C13	−179.8 (3)	N2—C31—C36—C35	−179.0 (3)
C12—S1—C15—C20	174.3 (3)	C34—S2—C37—C42	−122.2 (3)
C12—S1—C15—C16	−6.2 (4)	C34—S2—C37—C38	64.7 (3)
C20—C15—C16—C17	−0.7 (5)	C42—C37—C38—C39	−1.0 (5)
S1—C15—C16—C17	179.9 (3)	S2—C37—C38—C39	172.0 (3)
C15—C16—C17—C18	−0.5 (6)	C37—C38—C39—C40	0.5 (6)
C16—C17—C18—C19	1.4 (5)	C38—C39—C40—C41	0.6 (6)
C16—C17—C18—C21	−179.2 (4)	C38—C39—C40—C43	−178.5 (4)
C17—C18—C19—C20	−1.1 (6)	C39—C40—C41—C42	−1.1 (5)
C21—C18—C19—C20	179.5 (4)	C43—C40—C41—C42	178.0 (3)
C18—C19—C20—C15	−0.1 (6)	C38—C37—C42—C41	0.4 (5)
C16—C15—C20—C19	1.0 (5)	S2—C37—C42—C41	−173.0 (3)
S1—C15—C20—C19	−179.5 (3)	C40—C41—C42—C37	0.7 (5)
C17—C18—C21—O2	−9.5 (6)	C39—C40—C43—O4	−172.4 (4)
C19—C18—C21—O2	169.9 (4)	C41—C40—C43—O4	8.5 (6)
C17—C18—C21—C22	170.0 (4)	C39—C40—C43—C44	7.1 (6)
C19—C18—C21—C22	−10.6 (6)	C41—C40—C43—C44	−172.0 (4)

### Hydrogen-bond geometry (Å, º)

Cg1, Cg2, Cg3 and Cg6 are the centroids of the C2–C7, C9–C14, C15–C20 and C37–C42 rings, respectively.

**Table:**

*D*—H···*A*	*D*—H	H···*A*	*D*···*A*	*D*—H···*A*
C17—H17···*Cg*6^i^	0.93	3.00	3.734 (4)	137
C26—H26···*Cg*1	0.93	2.96	3.763 (4)	146
C32—H32···*Cg*2	0.93	2.98	3.706 (4)	136
C41—H41···*Cg*3^ii^	0.93	2.99	3.670 (4)	131

Symmetry codes: (i) *x*+1, *y*+1, *z*; (ii) *x*−1, *y*, *z*.
