# Supplementary figures and images for: Crystal structure of (E)-1-[4-({4-[(4-meth­oxy­benzyl­idene)amino]­phen­yl}sulfan­yl)phen­yl]ethan-1-one
Source: Acta Crystallogr E Crystallogr Commun. 2015 Jan 14;71(Pt 2):o109–10. doi: 10.1107/S205698901500033X (PMC4384611; doi:10.1107/S205698901500033X)

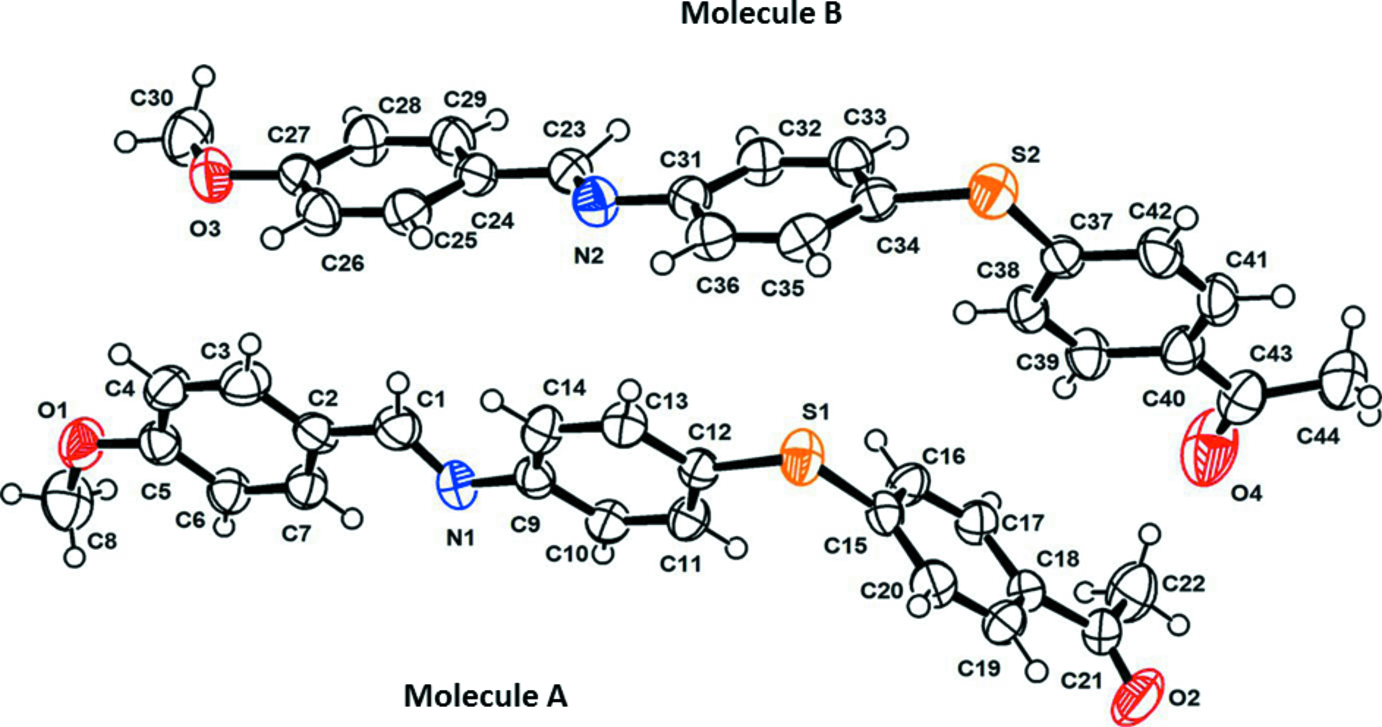

Supplement: Supplementary file 4 [file e-71-0o109-fig1.tif]

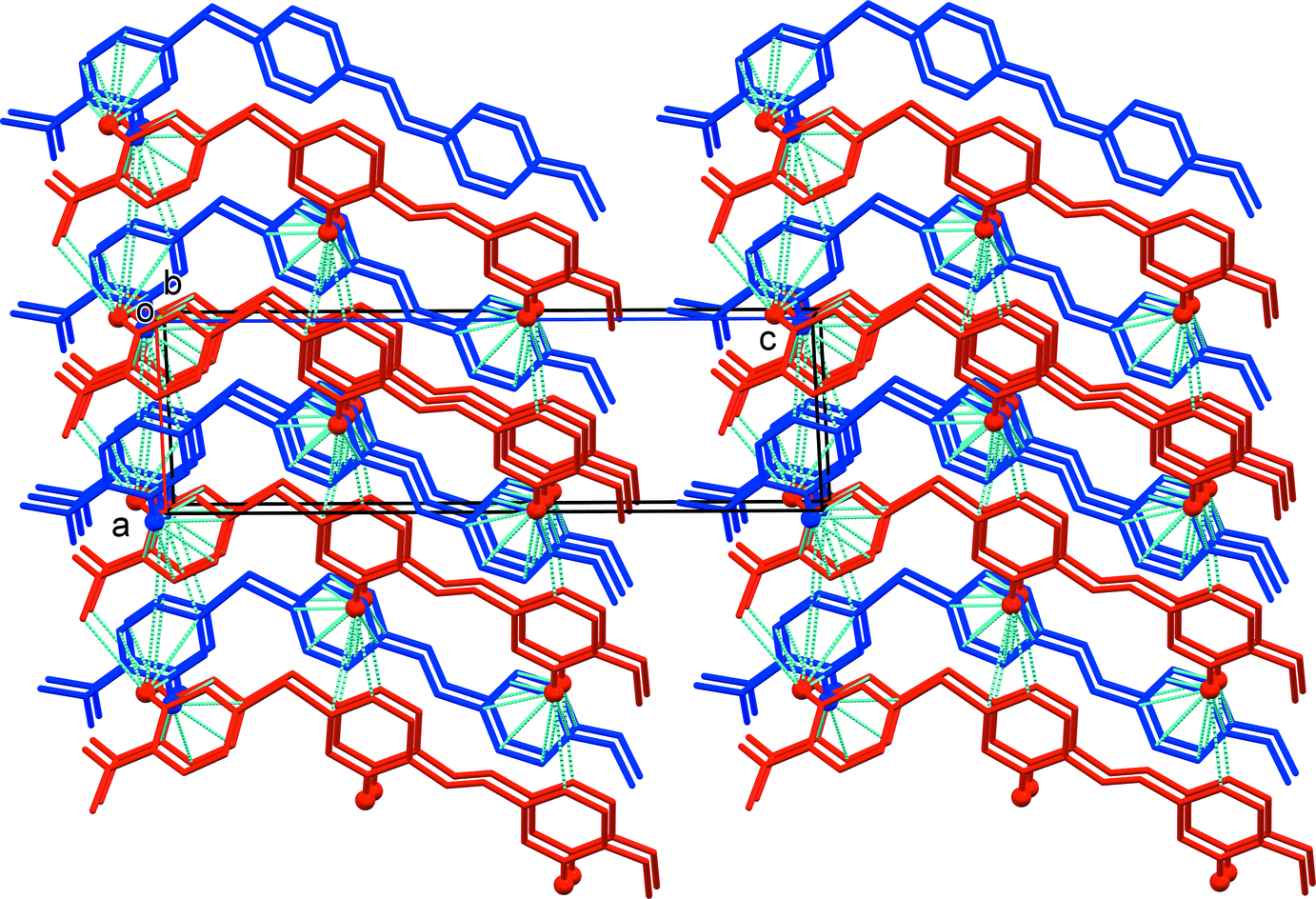

Supplement: Supplementary file 5 [file e-71-0o109-fig2.tif]
